# Alleviative effects of magnetic Fe_3_O_4_ nanoparticles on the physiological toxicity of 3-nitrophenol to rice (*Oryza sativa* L.) seedlings

**DOI:** 10.1515/biol-2022-0060

**Published:** 2022-06-15

**Authors:** Wangqing Sainao, Zhenzhen Shi, Hailong Pang, Hanqing Feng

**Affiliations:** College of Life Science, Northwest Normal University, 730070, Lanzhou, Gansu, China; New Rural Development Research Institute, Northwest Normal University, 730070, Lanzhou, Gansu, China

**Keywords:** alleviative effects, magnetic iron oxide nanoparticles, toxicity of 3-nitrophenol, rice seedlings

## Abstract

In the present study, we explored whether magnetic iron oxide nanoparticles (MNPs-Fe_3_O_4_) can be used to alleviate the toxicity of 3-nitrophenol (3-NP) to rice (*Oryza sativa* L.) seedlings grown under hydroponic conditions. The results showed that 3-NP from 7 to 560 μM decreased the growth, photochemical activity of the photosystem II (PS II), and chlorophyll content of the seedlings in a concentration-dependent manner. In the presence of 3-NP, 2,000 mg L^−1^ MNPs-Fe_3_O_4_ were added to the growth medium as the absorbents of 3-NP and then were separated with a magnet. The emergence of MNPs-Fe_3_O_4_ effectively alleviated the negative effects of 3-NP on rice seedlings. In addition, the long-term presence of MNPs-Fe_3_O_4_ (from 100 to 2,000 mg L^−1^) in the growth medium enhanced the growth, production of reactive oxygen species (ROS), activities of antioxidant enzymes, photochemical activity of PS II, and chlorophyll content of the rice seedlings. These results suggest that MNPs-Fe_3_O_4_ could be used as potential additives to relieve the physiological toxicity of 3-NP to rice seedlings.

## 1 Introduction

Nitrophenols (NPs) are widely used as the raw materials or intermediates for manufacturers of explosives, pharmaceuticals, pesticides, pigments, dyes, wood preservatives, and rubber chemicals [1]. As a result, NPs are abundantly presented in aquatic environments, including river water, wastewater, and industrial effluents [2]. Even at ultralow concentrations, these NPs still have potential toxicity to human beings and animals. Thus, the US Protection Agency (UPA) has listed NPs on its “Priority Pollutant List” [3].

NPs are also highly toxic to plants [4]. Previous works have found that NPs can cause DNA damage, increase oxidative stress, and decrease the contents of chlorophyll and auxin in plants. In the last decades, the threat of NPs to agriculture has deserved special attention since it has been reported that NPs are found in the irrigation water [5], and 0.7 mM NPs in the irrigation water can cause a large number of plants to reduce production [6]. Compared with animals, plants have less mobility and thus have to face environmental pollutants frequently. In fact, before obvious and visual alterations of morphology are observed, many physiological responses of plants to toxic pollutants have occurred and ultimately determined the fate of plants [7]. Therefore, it is important to develop effective technology to reduce the physiological toxicity of NPs to crops.

Direct removal of pollutants from the contaminated environment by adsorbents is considered an effective method to limit the toxicity of pollutants to plants and other organisms [8]. For example, pesticides have undesirable impacts on human health and may appear as pollutants in water sources. A variety of activated carbon materials have been used as adsorbents to remove different varieties of pesticides from water sources [9]. Besides activated carbon, nanoparticles are applied as efficient adsorbents to remove the pollutants from the waterbody [10,11], because nanoparticles have well dispersion in water and can be easily obtained [12]. However, applying common nanoparticles in aquatic or semiaquatic environments would inevitably cause secondary pollution. The magnetic iron oxide nanoparticles (MNPs-Fe_3_O_4_) are among the most widely applied nanomaterials in the industrial, biomedical, and biotechnological sectors [13]. MNPs-Fe_3_O_4_ have unique electric and magnetic properties based on the transfer of electrons between Fe^2+^ and Fe^3+^ in the octahedral sites [14]. Furthermore, compared to other nanomaterials, MNPs-Fe_3_O_4_ have exhibited better chemical stability, a large surface area, lower toxicity and price, and biocompatibility [15]. Thus, MNPs-Fe_3_O_4_ have been used as absorbents to remove contaminants from wastewater and can be easily separated from water by applying a magnetic field to avoid secondary pollution [15]. However, whether such properties of MNPs-Fe_3_O_4_ could be applied in limiting the toxicity of NPs to crops has not been extensively studied.

As one aquatic or semi-aquatic plant species, rice (*Oryza sativa* L.) has remarkable economic and alimentary importance [16]. In the present study, we develop a simple and novel method for alleviating the toxicity of 3-NP to rice seedlings. Briefly, MNPs-Fe_3_O_4_ as absorbents of 3-NP are added into the growth medium of rice seedlings. The emergence of MNPs-Fe_3_O_4_ can effectively alleviate the negative effects of NP on rice seedlings, and the MNPs-Fe_3_O_4_ can be separated from the growth medium by the magnetic field. We also further study the physiological responses of rice seedlings to the long-term presence of MNPs-Fe_3_O_4_ to evaluate the potential effects of MNPs-Fe_3_O_4_ on the plants. We believe that this research can provide some references on reducing the toxicity of NPs to crops and can further enrich current knowledge about the application of MNPs-Fe_3_O_4_ in the botany field.

## 2 Materials and methods

### 2.1 Materials

**Figure A1:**
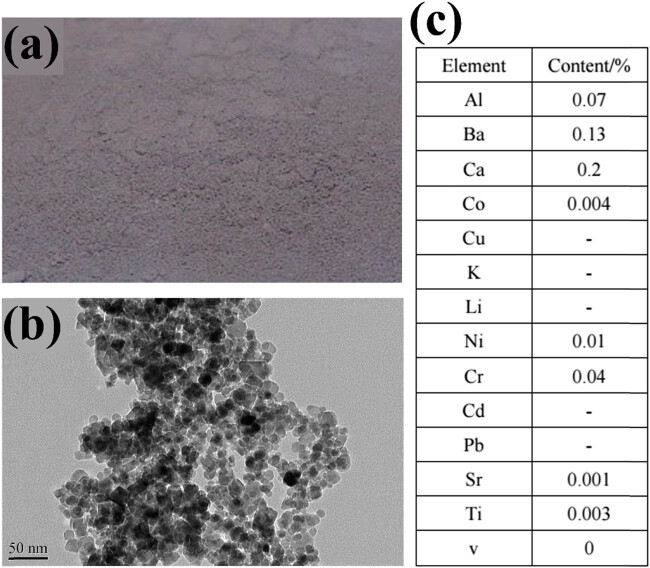
The characterization of MNPs-Fe_3_O_4_ used in this study. (a) Photo-images of MNPs-Fe_3_O_4_. (b) Transmission-electron micrographs of the MNPs-Fe_3_O_4_. (c) Elemental analysis of MNPs-Fe_3_O_4_.

Seeds of rice (Jing-you No. 957) were obtained from China National Seed Group Company, and MNPs-Fe_3_O_4_ were obtained from China Nanjing Emperor Nano Material Company. The purity of MNPs-Fe_3_O_4_ is >99.5%, and the main impurity of the MNPs-Fe_3_O_4_ is barium. The diameter and surface of MNPs-Fe_3_O_4_ are 14.1 nm and 81.98 m^2^ g^−1^, respectively (Figure A1).

### 2.2 Treatments

Rice seeds were surface-sterilized for 2 min by sodium hypochlorite solution (2%, v/v). After then, the seeds were thoroughly rinsed with distilled water for 30 min and placed in Petri dishes for germination in the dark at 25°C. The germinated seeds with primary root (at least 0.5 mm) were sown in the beakers and were irrigated with 1/2 strength of Hoagland’s nutrient solution. The seedlings were grown in a climate room at 27/23°C day/night temperatures and a 12/12 h light/dark regime with 130 μmol (photon) m^−2^ s^−1^ photosynthetically active radiation (PAR). The nutrient solution in the beakers was replaced with freshly prepared 1/2 strength Hoagland’s nutrient solution every 7 days.

In the first set of experiments, 3-NP was dissolved with 3 mL of ethanol (95%, v/v) and was diluted with distilled water to the concentrations required. Twenty-one-day-old seedlings received a 70, 140, 280, and 560 μM NP solution and were cultivated under the above conditions for 5 days. The seedlings that received the solvent (an ethanol solution with a concentration equivalent to that in the NP solution) alone were set as the treatment with 0 μM NP (controls).

**Figure A2:**
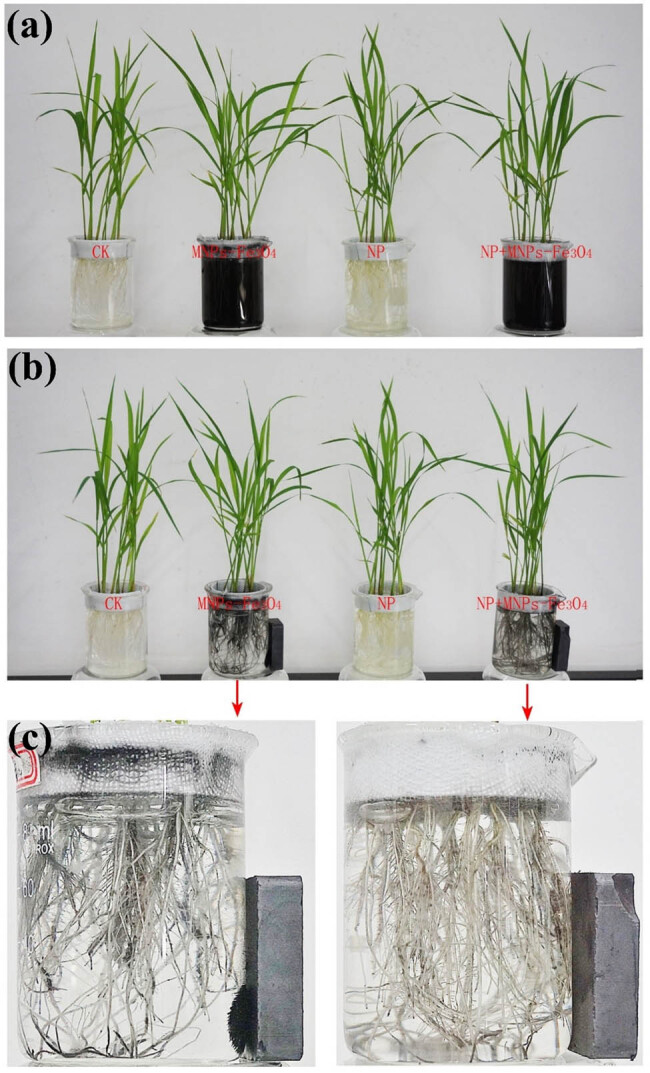
The scheme of experimental processing for evaluating the alleviative effects of MNPs-Fe_3_O_4_ on toxicity of NP to rice seedlings. (a) The rice seedlings were subjected to different treatments. CK: controls (the seedlings that received the solvent of NP solution only); MNPs-Fe_3_O_4_: the seedlings that received 2,000 mg L^−1^ MNPs-Fe_3_O_4_; NP: the seedlings that received 280 µM NP; NP + MNPs-Fe_3_O_4_: the seedlings that received 280 µM NP plus 2,000 mg L^−1^ MNPs-Fe_3_O_4_. (b) After the rice seedlings were treated as described above, MNPs-Fe_3_O_4_ were separated with magnet. (c) The enlarged image of magnetic separation of MNPs-Fe_3_O_4_ by magnet.

In the second set of experiments, 21-day-old seedlings were subjected to different treatments. In NP, the seedlings received 280 μM NP and were maintained under the above conditions for 5 days. In NP + MNPs-Fe_3_O_4_ or MNPs-Fe_3_O_4_, the seedlings received 280 μM NP or the solvent alone and were then maintained under the above conditions for 5 h. Afterward, MNPs-Fe_3_O_4_ with the final concentration of 2,000 mg L^−1^ were added to the beaker, and the culture suspension was ultrasonically dispersed (100 W, 40 Hz) for 30 min. After then, a magnet was placed aside for the magnetic separation of MNPs-Fe_3_O_4_ (Figure A2). After the separation, the magnet was removed, and the seedlings were cultivated for 5 days under the above conditions. In CK (controls), the seedlings received the solvent alone (i.e., 0 μM NP) and were maintained under the above conditions for 5 days.

In the last set of experiments, MNPs-Fe_3_O_4_ were added into the freshly prepared 1/2 strength Hoagland’s nutrient solution with the final concentrations of 100, 500, 1,000, and 2,000 mg L^−1^, respectively. The freshly prepared 1/2 strength Hoagland’s nutrient solution without MNPs-Fe_3_O_4_ was set as the CK (i.e., 0 mg L^−1^ MNPs-Fe_3_O_4_). The MNPs-Fe_3_O_4_ suspensions and freshly prepared Hoagland’s nutrient solution above were ultrasonically dispersed (100 W, 40 Hz) for 1 h and were used to replace the Hoagland’s nutrient solution in the beakers, in which rice seedlings had grown for 5 days. The seedlings were grown in the MNPs-Fe_3_O_4_ suspension or 1/2 strength of Hoagland’s nutrient solution for 21 days.

### 2.3 Measurement of the growth of the seedlings

The height of the seedlings was measured as described by Lin et al. [17], and the length of roots of the seedlings was measured as described by Elise et al. [18] by using Image-J. To measure dry weight, the seedlings were washed with distilled water 5–6 times to remove any medium and particles attached to the plant surfaces and then wiped with a sterile filter paper to remove the excess liquid. Afterward, the samples were first dried for 30 min at 100°C and then oven-dried at 70°C until constant weights. After then, the dry weight of above-ground parts, dryweight of below-ground parts, and dry weight of the whole seedlings were determined [19].

### 2.4 Measurement of chlorophyll content and chlorophyll fluorescence

Chlorophyll content was measured according to the method described by Zhang et al. [20] with some modifications. The leaves were homogenized with 4 mL of 80% (v/v) acetone, and the homogenate was then centrifuged at 13,000×*g* for 20 min at 4°C. The absorbance of the supernatant was measured at 645 and 663 nm.

Chlorophyll fluorescence parameters were measured according to the method of Zhang et al. [20] by using a portable fluorometer (PAM-2500, Walz, Germany). Y(II) (the effective photochemical quantum yield of PS II) was defined as (Fm′ − Fs)/Fm′, where Fm′ is the maximum fluorescence emission from the light-adapted state measured with a pulse of saturating light, and Fs is the steady-state level of fluorescence emission at the given irradiance. The photochemical quenching (qP) was defined as (Fm′ − Fs)/(Fm′ − Fo′), where Fo′ is the minimal fluorescence of the light-adapted state measured with a far-red pulse. The photosynthetic electron transport rate (ETR) through PS II was calculated as Y(II) × PAR × 0.5 × 0.84.

### Measurement of the hydrogen peroxide (H_2_O_2_) content and the production rate of superoxide anion ( {\text{O}}_{2}^{-}] ) of roots

2.5

The measurement of H_2_O_2_ content was performed according to the method of Li et al. [21]. Roots (40 mg) were ground with 0.1% (w/v) trichloroacetic acid, and the homogenate was centrifuged at 12,000×*g* for 10 min. After then, 0.7 mL of the supernatant was mixed with 0.7 mL of 10 mM phosphate-buffered solution (PBS, pH 7.0) and 1.4 mL of 1 M KI. The absorbance of the solution was measured at 390 nm. H_2_O_2_ contents were calculated using a standard curve prepared with the known concentrations of H_2_O_2_.

For the measurement of the production rate of {\text{O}}_{2}^{-}] , roots (50 mg) were incubated in 3 mL of the reaction mixture containing 50 mM Tris-HCl buffer (pH 6.5), 0.2 mM nitro-blue tetrazolium (NBT), 0.2 mM NADH, and 250 mM sucrose for 24 h at room temperature in the dark. The absorbance of the blue mono-formazan formed was measured at 530 nm and its concentration was calculated according to the method of Achary et al. [22].

### 2.6 Antioxidant enzyme activities

The roots (50 mg) were ground with 1 mL of chilled 50 mM PBS (pH 7.8) containing 0.1 mM ethylenediaminetetraacetic acid (EDTA) and 1% polyvinylpyrrolidone. The homogenate was centrifuged at 12,000×*g* for 30 min, and the supernatant (as enzyme extraction) was collected to determine the activities of antioxidant enzymes [21].

The superoxide dismutase (SOD) activity was measured by the method of Dhindsa and Matowe [23] with some modifications. The reaction mixture consisted of 50 mM PBS (pH 7.6), 13 mM methionine, 75 mM NBT, 0.1 mM EDTA-Na_2_, and an appropriate amount of enzyme extraction. The reaction was started by the addition of 2 mM lactochrome. After illumination for 30 min at 25°C, the absorbance was recorded at 560 nm. The activity of SOD was expressed in unit mg^−1^ of fresh weight (Fw).

The peroxidase (POD) activity was measured following the method of Rao et al. [24] with some modifications. The enzyme extraction was mixed with 3 mL of the reaction medium containing 50 mM PBS (pH 7.0) and 20 mM guaiacol. After incubation at 25°C for 5 min, 6 mM H_2_O_2_ was added to initiate the reaction. The absorbance changes at 470 nm within 2 min were recorded to calculate the POD activity. The POD activity was expressed in ΔOD_470_ min^−1^ mg^−1^ Fw.

The catalase (CAT) activity was measured by the method of Zhang et al. [20] with some modifications. The enzyme extraction was added to 3 mL of 50 mM PBS (pH 7.0) and incubated at 25°C for 5 min. After that, the reaction was started by adding 6 mM H_2_O_2_, and the absorbance changes were recorded at 240 nm for 2 min. The CAT activity was expressed in ΔOD_240_ min^−1^mg^−1^ Fw.

For the measurement of ascorbate peroxidase (APX) activity, the roots (20 mg) were ground with 1 mL of 50 mM PBS (pH 7.0) containing 1 mM EDTA-Na_2_ and 1 mM ascorbate (ASA). After centrifugation for 20 min at 10,000×*g*, the supernatant was collected to measure the APX activity according to the method of Nakano and Asada [25] with some modifications. The assay was carried out in a reaction mixture consisting of 50 mM PBS (pH 7.0), 0.5 mM ASA, 3 mM H_2_O_2_, and the right amount of enzyme extraction. The changes in the absorbance at 290 nm were recorded at 25°C for 2 min after the addition of H_2_O_2_. The APX activity was expressed in ΔOD_290_ min^−1^ mg^−1^ Fw.

### 2.7 Statistical analysis

The data are expressed as the mean ± standard deviation (SD) of at least three independent replicates. Statistical analysis was evaluated with *t*-test methods. *P* < 0.05 was considered statistically significant.

## 3 Results

### 3.1 The effects of NP on the growth of the seedlings

The level of the growth of the seedlings was expressed by measuring the seedling height (SH), length of root (LR), dry weight of below-ground part (DWB), dry weight of above-ground part (DWA), and dry weight of whole seedlings (DWW). As shown in Table 1, the stress of NP decreased the values of SH, LR, DWB, DWA, and DWW. When the concentration of NP reached 280 µM, SH, LR, DWB, DWA, and DWW were significantly decreased by 37.4, 25.7, 27.3, 24.5, and 15.9%, respectively, compared to the controls. The decreases in these parameters were further aggravated after the seedlings were treated with 560 µM NP.

**Table 1:** The effects of NP on SH, LR, DWB, DWA, and DWW

NP concentrations (µM)	SH (mm)	LR (mm)	DWA (g)	DWB (g)	DWW (g)
0	142.233 ± 9.899^a^	88.181 ± 12.310^a^	0.049 ± 0.0016^a^	0.022 ± 0.0019^a^	0.063 ± 0.0015^a^
70	134.999 ± 11.833^ab^	83.450 ± 11.707^ab^	0.045 ± 0.0022^ab^	0.021 ± 0.0015^a^	0.061 ± 0.0021^a^
140	122.705 ± 17.187^b^	75.929 ± 8.557^b^	0.042 ± 0.0010^b^	0.018 ± 0.0011^b^	0.059 ± 0.0017^a^
280	89.086 ± 7.791^c^	65.491 ± 4.629^c^	0.037 ± 0.0017^c^	0.016 ± 0.0005^c^	0.053 ± 0.0011^b^
560	54.839 ± 4.256^d^	61.879 ± 6.268^c^	0.021 ± 0.0014^d^	0.012 ± 0.0016^d^	0.035 ± 0.0044^c^

Each value represents the mean ± SD of three individual replications at least.

Different small letters in superscript indicate a significant difference (at *P* < 0.05 levels) of the same parameter among the seedlings treated with different concentrations of NP.

### 3.2 Effect of NP on chlorophyll fluorescence parameters and chlorophyll content of the seedlings

As shown in Table 2, the values of the effective quantum yield of PSII photochemistry (Y(II)), photochemical quenching (qP), electron transport rate (ETR), and chlorophyll content were decreased with the increase in NP concentrations. The chlorophyll content was also decreased with the increase in NP concentrations. Compared to the controls, the chlorophyll content was decreased by 15.97, 25.91, 39.29, and 48.69%, respectively, after treatment with 70, 140, 280, and 560 µM NP (Table 2).

**Table 2:** Effect of NP stress on the Y(II), qP, ETR, and chlorophyll content of the rice seedlings

NP concentrations (µM)	Chlorophyll fluorescence parameters			Chlorophyll content (mg g^−1^ Fw)
	Y(II)	qP	ETR	
0	0.361 ± 0.006^a^	0.747 ± 0.037^a^	24.012 ± 0.428^a^	2.980 ± 0.114^a^
70	0.365 ± 0.011^a^	0.739 ± 0.033^a^	24.308 ± 0.765^a^	2.504 ± 0.129^b^
140	0.317 ± 0.005^b^	0.605 ± 0.036^b^	18.679 ± 1.034^b^	2.208 ± 0.158^c^
280	0.251 ± 0.008^c^	0.431 ± 0.045^c^	16.619 ± 0.320^c^	1.809 ± 0.053^d^
560	0.156 ± 0.025^d^	0.399 ± 0.028^c^	11.527 ± 0.493^d^	1.529 ± 0.111^e^

Each value represents the mean  ±  SD of three individual replications at least. Different small letters in superscript indicate a significant difference (at *P <* 0.05) of the same parameter among the seedlings treated with different concentrations of NP.

### 3.3 Effects of MNPs-Fe_3_O_4_ on the growth of the seedlings under NP stress

We further studied whether the MNPs-Fe_3_O_4_ in the growth medium can alleviate NP-induced physiological stresses to rice seedlings. We concentrated on the seedlings subjected to 280 μM NP, since NP at this concentration caused the significant changes in all parameters measured (Tables 1 and 2).

**Figure 1:**
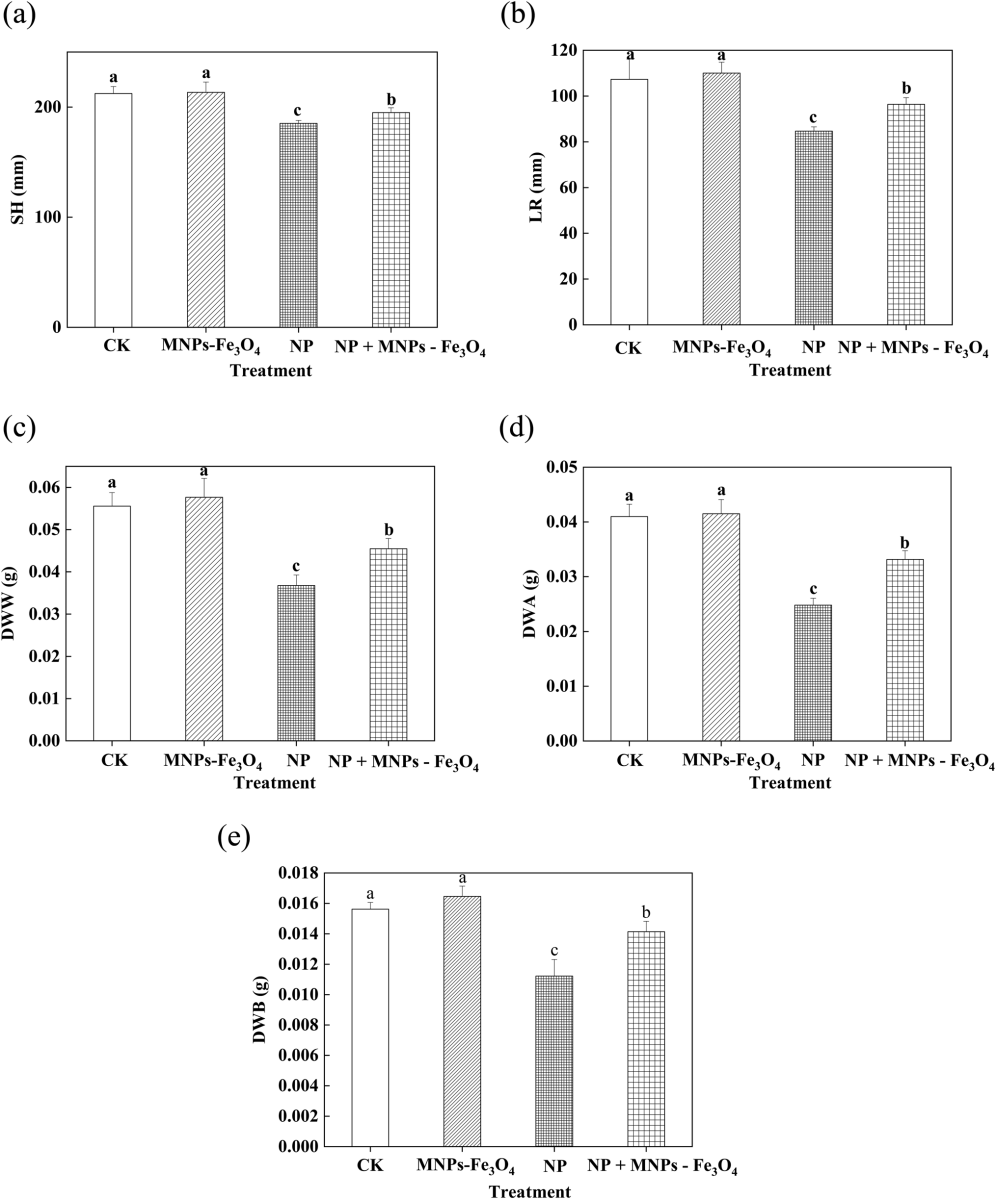
Effects of MNPs-Fe_3_O_4_ on the SH (a), LR (b), DWW (c), DWA (d), and DWB (e) under NP stress. CK: treatment with received the solvent alone as the controls; MNPs-Fe_3_O_4_: treatment with magnetic Fe_3_O_4_ nanoparticles (2,000 mg L^−1^); NP: treatment with NP (280 µM); and NP + MNPs-Fe_3_O_4_: treatment with a combination of NP (280 µM) and magnetic (Fe_3_O_4_) nanoparticles (2,000 mg L^−1^). Each value represents the mean  ±  SD of three individual replications at least. Different small letters indicate a significant difference (at *P* < 0.05) among the different treatments.

The growth of rice seedlings was significantly reduced after the treatment with 280 µM NP, whereas the presence of MNPs-Fe_3_O_4_ in the growth medium effectively alleviated the NP-induced reduction of growth of the seedlings. This was reflected by the observations that the value of SH, LR, DWW, DWA, and DWB of the seedlings exposed to NP + MNPs-Fe_3_O_4_ was significantly increased by 1.05-, 1.14-, 1.24-, 1.34-, and 1.26-fold, respectively, compared to the seedlings exposed to NP alone (Figure 1).

### 3.4 Effects of MNPs-Fe_3_O_4_ on the photochemical activity of PS II and chlorophyll content of the seedlings under NP stress

**Figure 2:**
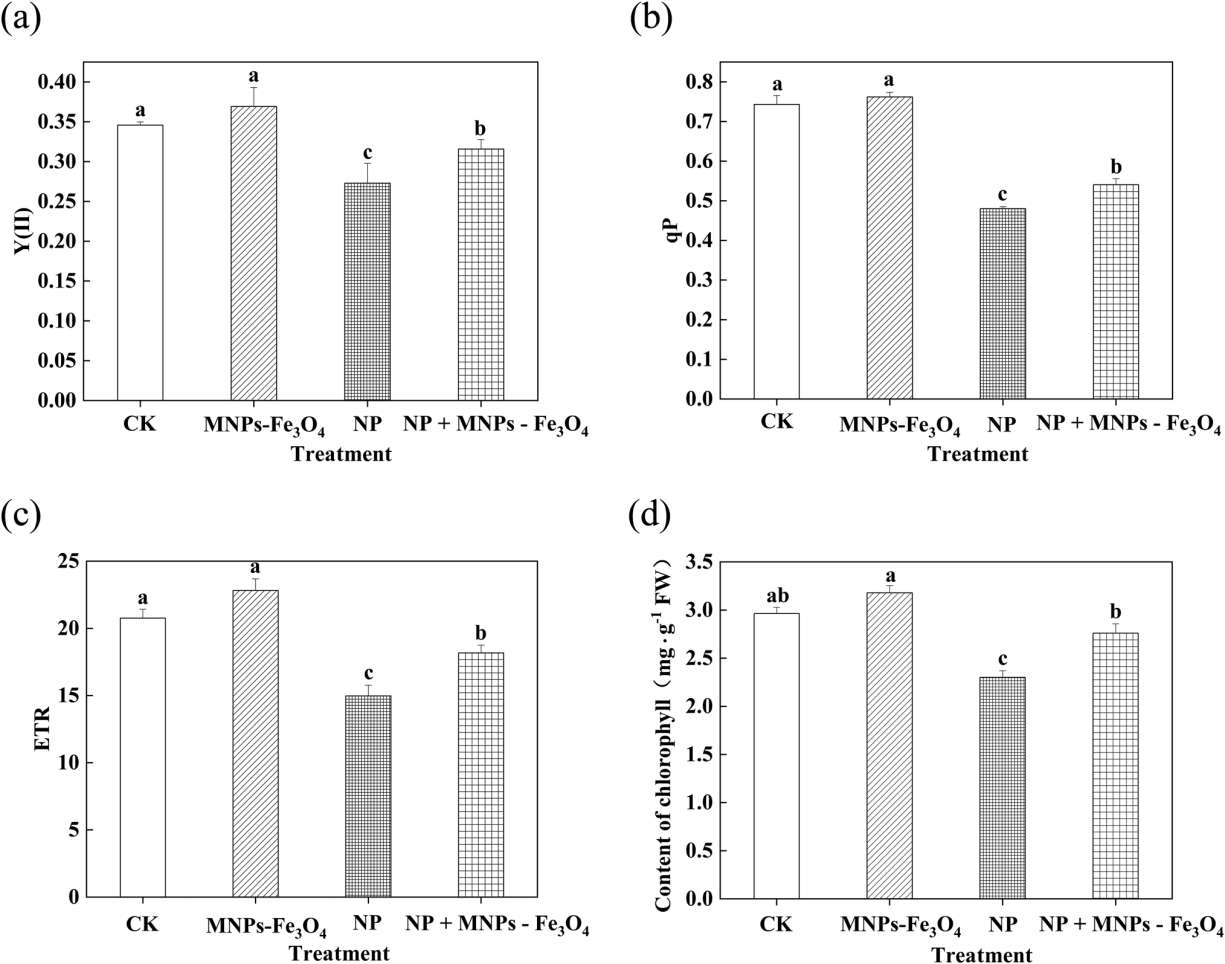
Effects of MNPs-Fe_3_O_4_ on the Y(II) (a), qP (b), ETR (c), and chlorophyll content (d) of the rice seedlings under NP stress. CK: treatment with received the solvent alone as the controls; MNPs-Fe_3_O_4_: treatment with magnetic Fe_3_O_4_ nanoparticles (2,000 mg L^−1^); NP: treatment with NP (280 µM); and NP + MNPs-Fe_3_O_4_: treatment with combination of NP (280 µM) and magnetic (Fe_3_O_4_) nanoparticles (2,000 mg L^−1^). Each value represents the mean  ±  SD of three individual replications at least. Different small letters indicate a significant difference (at *P* < 0.05) among the different treatments.

Compared with the controls, the values of Y(II), qP, ETR, and chlorophyll content were significantly decreased under NP stress. However, the values of Y(II), qP, ETR, and chlorophyll content of the seedlings were significantly increased by the treatment with NP + MNPs-Fe_3_O_4_, compared with the seedlings exposed to the NP treatment alone (Figure 2) (Y(II), qP, ETR, and chlorophyll content were increased by 1.16-, 1.13-, 1.21-, and 1.19-fold, respectively).

### 3.5 Effects of long-term presence of MNPs-Fe_3_O_4_ on the growth of the seedlings

**Figure 3:**
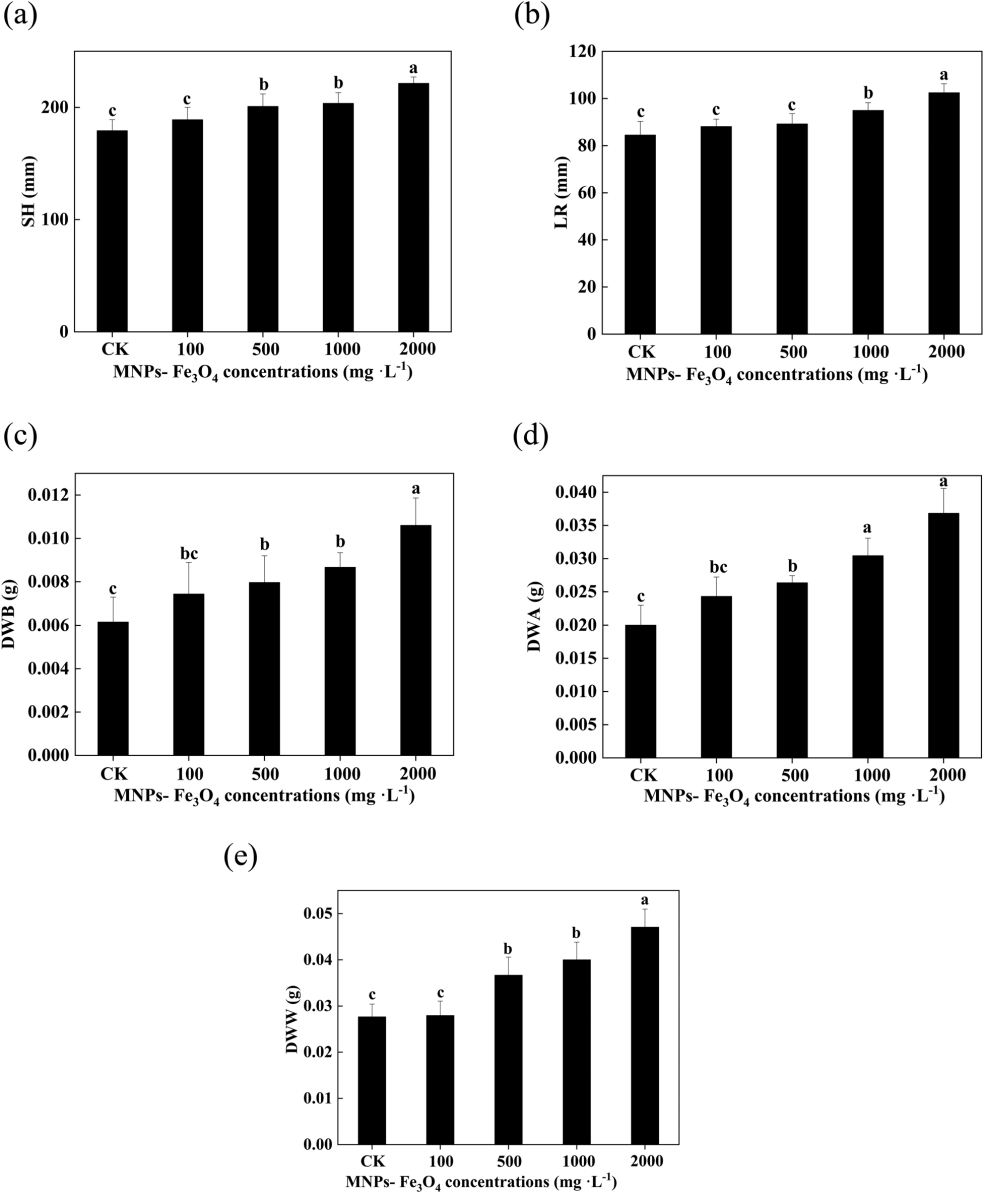
Effects of MNPs-Fe_3_O_4_ on the SH (a), LR (b), DWB (c), DWA (d), and DWW (e). Each value represents the mean  ±  SD of three individual replications at least. Different small letters indicate a significant difference (at *P* < 0.05) among the seedlings treated with different concentrations of MNPs-Fe_3_O_4_.

In comparison with the controls, treatment with 100 mg L^−1^ MNPs-Fe_3_O_4_ did not significantly affect the growth of the seedlings. With the further increase in concentrations of MNPs-Fe_3_O_4_, the values of SH, LR, DWB, DWA, and DWW were gradually increased (Figure 3). Treatment with 2,000 mg L^−1^ MNPs-Fe_3_O_4_ caused the most noticeable increase in seedling growth. Compared to the controls, the values of SH, LR, DWB, DWA, and DWW were increased by 1.24-, 1.21-, 1.84-, 1.72-, and 1.70-fold, respectively, after treatment with 2,000 mg L^−1^ MNPs-Fe_3_O_4_.

### 3.6 Effects of MNPs-Fe_3_O_4_ on the production of reactive oxygen species (ROS) and activities of antioxidant enzymes of the seedling roots.

**Figure 4:**
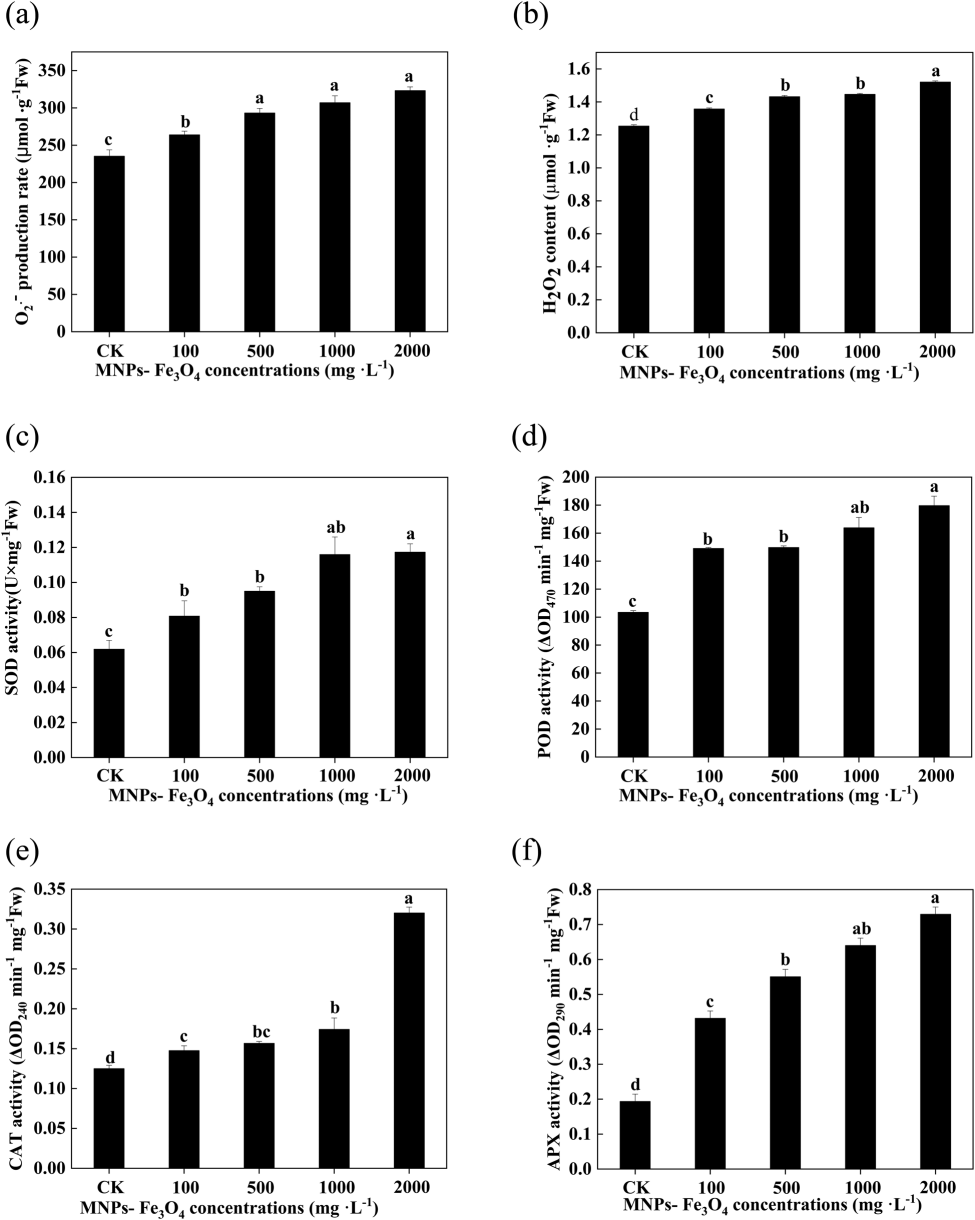
Effects of MNPs-Fe_3_O_4_ on the production rate of superoxide anion ( {\text{O}}_{2}^{-}] ) (a), hydrogen peroxide (H_2_O_2_) content (b), and the activities of SOD (c), POD (d), CAT (e), and APX (f) of rice seedling roots. Each value represents the mean  ±  SD of three individual replications at least. Different small letters indicate a significant difference (at *P* < 0.05) among the seedlings treated with different concentrations of MNPs-Fe_3_O_4_.

The production rate of {\text{O}}_{2}^{-}] and the content of H_2_O_2_ in the seedling roots were significantly increased with the increase in concentrations of MNPs-Fe_3_O_4_ (Figure 4a and b). We also investigated the effects of MNPs-Fe_3_O_4_ on the activities of the antioxidant enzymes, including SOD, POD, CAT, and APX. The results showed that the activities of SOD, POD, CAT, and APX were significantly increased by the treatment with 100–2,000 mg L^−1^ MNPs-Fe_3_O_4_ (Figure 4c–f).

### 3.7 Effects of MNPs-Fe_3_O_4_ on the photochemical activity of PS II and chlorophyll content of the seedlings

**Figure 5:**
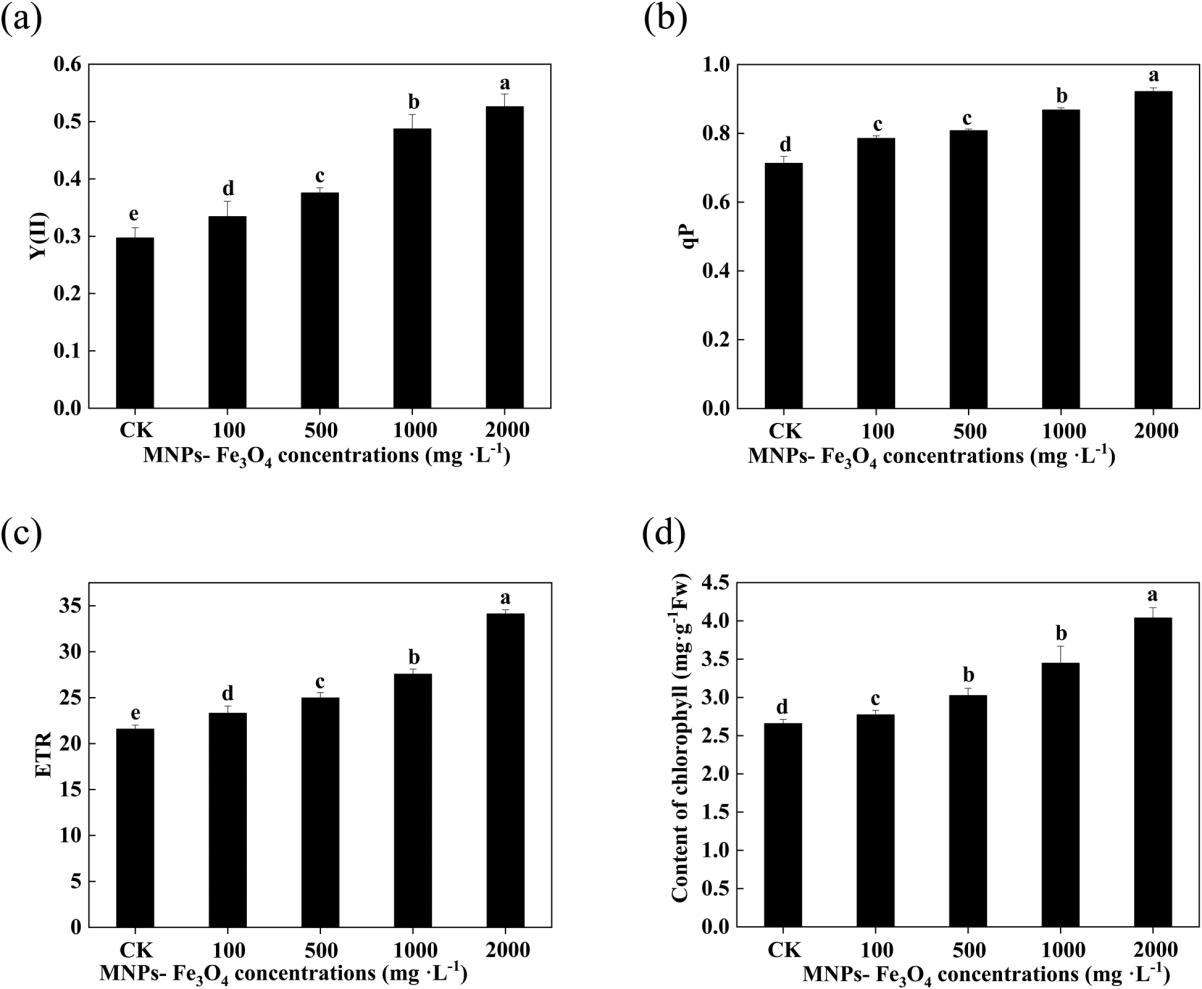
Effects of MNPs-Fe_3_O_4_ on Y(II) (a), qP (b), ETR (c), and chlorophyll content (d) of the rice seedlings. Each value represents the mean  ±  SD of three individual replications at least. Different small letters indicate a significant difference (at *P* < 0.05) among the seedlings treated with different concentrations of NP.

Treatment with 100–2,000 mg L^−1^ MNPs-Fe_3_O_4_ significantly increased the values of Y(II), qP, and ETR of the leaves of the seedlings (Figure 5a–c). Simultaneously, the chlorophyll content was also increased with the increase in concentrations of MNPs-Fe_3_O_4_ (Figure 5d).

## 4 Discussion

It is well known that organic contaminants can inhibit the plant growth [26]. In the present study, the growth of rice seedlings was significantly inhibited by NP stress (Table 1). Photosynthesis is one of the most important metabolic processes of plants. The primary step of photosynthesis is to absorb light and transfer excitation energy to the reaction centers of PSII to drive the primary photochemical reactions. Measurement of chlorophyll fluorescence provides a rapid and sensitive mean for detecting the photochemical activity of PSII [27,28]. The inhibition of growth by NP stress was observed to be followed by decreases in Y(II), ETR, qP, and chlorophyll content (Table 2). These observations are in agreement with the findings of Zhang et al. [29] and Liu et al. [30], who revealed that NPs could reduce the growth, photosynthesis, and biosynthesis of chlorophyll in plants.

Although the application of nanoparticles as adsorbents provides a convenient method to remove the contaminants from the waterbody, the accumulation and remnants of the nanoparticles could lead to potential contamination [31]. Compared to common nanomaterials, the magnetic properties of MNPs-Fe_3_O_4_ allow easy separation of the nanoparticles from water in the presence of an external magnetic field [15]. Therefore, in the present work, MNPs-Fe_3_O_4_ were employed as the absorbents of NP and then were separated with magnet rice seedlings. Under NP stress, such application of MNPs-Fe_3_O_4_ significantly alleviated the NP-induced adverse effects on growth, photochemical reactions, and biosynthesis of chlorophyll of rice seedlings (Figures 1 and 2). Thus, it is suggested that MNPs-Fe_3_O_4_ may reduce the accumulation of NP in the seedlings by absorbing NP, thus effectively alleviating the toxicity of NP to the seedlings.

Although significant amounts of MNPs-Fe_3_O_4_ were separated from the growth medium through a magnet, small amounts of MNPs- Fe_3_O_4_ were still left in the growth medium (Figure A2). Thus, one can speculate that the residual MNPs-Fe_3_O_4_ could negatively affect the plants. Hence, to address such concern for environmental biosafety, we further evaluated the effects of long-term presence (during 21 days of growth of rice seedlings) of MNPs-Fe_3_O_4_ on the rice seedlings. The results showed that MNPs-Fe_3_O_4_ could enhance the growth, increase the ROS production and activities of antioxidant enzymes, and improve the photochemical activity of PS II and chlorophyll content (Figures 3–5). Such observations indicate that MNPs-Fe_3_O_4_ can limit the toxicity of NP to plants and improve the growth of plants.

Although the MNPs-Fe_3_O_4_ are not endogenous substances of plants, many reports available showed that MNPs-Fe_3_O_4_ could exert multiple positive effects on plants. It has been found that the dissolved iron ions from MNPs-Fe_3_O_4_ can increase the supply of Fe-ion, which is an advantageous component for plant growth and is required for many physiological processes of plants, such as chlorophyll biosynthesis [32,33,34]. Other works reported that the application of MNPs-Fe_3_O_4_ can enhance the absorption of other nutrients elements, such as calcium, potassium, and magnesium, in plants [35,36,37]. It is well known that ROS can act as important signal molecules in positively regulating plant growth, and antioxidant enzymes can protect the plant cell from damage from external environments [38]. In agreement with our results (Figure 4), many works revealed that MNPs-Fe_3_O_4_ could increase ROS production and stimulate the activities of antioxidant enzymes [39,40]. Thus, MNPs-Fe_3_O_4_ are thought to motivate the variations in plants’ defense mechanisms in response to environmental stresses by modulating ROS production and antioxidant status [41].

In theory, besides MNPs-Fe_3_O_4_, other types of magnetic nanoparticles can be used as additives to relieve the NP-induced stress on plants and can be separated by the magnetic field. However, it should be noted that different magnetic nanoparticles could have different effects on plants. For example, Tombuloglu et al. [42] found that manganese ferrite (MnFe_2_O_4_) nanoparticles at higher concentrations (250 or 1,000 mg L^−1^) remarkably decreased the dry weight of the leaf of barley (*Hordeum vulgare* L.) seedlings. In comparison, our present work showed that MNPs-Fe_3_O_4_ at higher concentrations (500–2,000 mg L^−1^) enhanced the dry weight of the below-ground part of rice seedlings. Such discrepancy may be attributed to the presence of Mn in the MnFe_2_O_4_ nanoparticles that could exert additional effects on the plants when higher concentrations of MnFe_2_O_4_ nanoparticles were applied since Mn is one of the heavy metals that can be harmful to plants at excessive levels [43]. Thus, the composition of elements in magnetic nanoparticles should be considered an important factor for more secure application, especially when a high dose of magnetic nanoparticles would be used.

In conclusion, the present result showed that the NP stress decreased the growth, photochemical activity of PS II, and chlorophyll content of the rice seedlings. Such toxicity of NP to the seedlings can be effectively alleviated by the addition of MNPs-Fe_3_O_4_ into the growth medium, followed by separation *via* the magnetic field. Furthermore, the long-term presence of MNPs-Fe3O4 in the growth medium positively affected the seedlings. These results indicate that MNPs-Fe_3_O_4_ could be used as a potential additive to relieve the NP-induced toxicity to plants. Future studies are expected to reveal whether MNPs-Fe_3_O_4_ could be used to cope with the simultaneous threat of inorganic and organic contaminants to plants. Moreover, research on biochemical and molecular levels is also needed for a deeper insight to understand the mechanism for the effects of MNPs-Fe_3_O_4_ on plants.

## Figures and Tables

**Figure A3:**
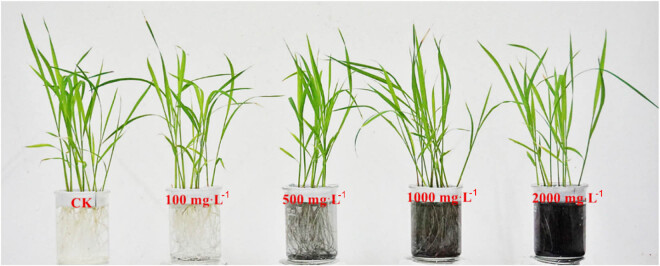
The scheme of experimental processing for evaluating the effects of MNPs-Fe_3_O_4_ at different concentrations on rice seedlings. Red letters indicate that the final concentration that was received by the rice seedlings. CK: the seedlings without treatment with MNPs-Fe_3_O_4_ were used as the controls).
